# Emerging Horizons: The Confluence of Artificial Intelligence and Graphic Medicine in Healthcare Education and Dentistry

**DOI:** 10.7759/cureus.90461

**Published:** 2025-08-19

**Authors:** Debanwita Dutta, Anjana Raut, Arun K Mohanty, Swati Samantaray, Ipsita Roy

**Affiliations:** 1 Prosthodontics and Crown and Bridge, Kalinga Institute of Dental Sciences, KIIT Deemed to be University, Bhubaneswar, IND; 2 Prosthodontics, Kalinga Institute of Dental Sciences, KIIT Deemed to be University, Bhubaneswar, IND; 3 Department of Humanities, School of Liberal Studies, KIIT Deemed to be University, Bhubaneswar, IND; 4 Orthodontics and Dentofacial Orthopaedics, Kalinga Institute of Dental Sciences, KIIT Deemed to be University, Bhubaneswar, IND

**Keywords:** artificial intelligence, clinical decision support, dental informatics, dentistry, graphic medicine, health literacy, patient education

## Abstract

Graphic medicine, the use of visual storytelling to convey health-related information, is gaining recognition in dentistry for its ability to enhance patient understanding and engagement. With the advent of artificial intelligence (AI), this medium is being significantly enriched to support both educational and clinical objectives. This review aimed to assess the integration of AI into graphic medicine within dentistry, focusing on its applications in patient education, clinical decision-making, and professional training. This narrative review synthesizes current literature and technological developments regarding AI tools such as natural language processing, image recognition, and generative design-and their implementation in creating personalized and culturally appropriate graphic content for dental care. AI-driven graphic medicine tools enable the creation of adaptive, user-responsive visual materials that enhance communication about oral hygiene, dental procedures, and post-treatment care. These tools support real-time patient interaction and improve the clarity, accessibility, and inclusivity of health information, especially for children, geriatric patients, and individuals with limited health literacy or cognitive challenges. The integration of AI into graphic medicine represents a transformative advancement in dental practice. It holds substantial promise for improving patient outcomes by facilitating personalized education and enhancing clinical workflows.

## Introduction and background

Graphic medicine refers to the concept of using graphic illustrations in the form of comics to communicate stories related to a disease, or a state of illness, caregiving, and easy anatomical illustrations of complex structures by medical students. Traditionally, graphic medicine was mainly used in medical emergency scenarios and to explain persistent deep-seated diseases where challenging situations are encountered regularly and have to be managed readily with a more comprehensible interaction between the medical staff and the patient attendants. British physician and artist Ian Williams first used the phrase "graphic medicine" to refer to the application of comics in medical settings [1]. Williams highlights the connection between graphic medicine and narrative medicine's goals in the groundbreaking work Graphic Medicine Manifesto, writing: "graphic medicine combines the principles of narrative medicine with an exploration of the visual systems of comic art, interrogating the representation of physical and emotional signs and symptoms within the medium" [2].

Three graphic memoirs by women - Cancer Vixen by Marisa Acocella Marchetto, Tangles by Sarah Leavitt, and Not Funny Ha-Ha by Leah Hayes - have been examined in order to investigate the usage of the graphic form in the narration of a disease experience. These works do not merely document disease experiences; they actively reframe the illness journey through a gendered, resistive lens. This study also suggests that by rejecting a straight line from diagnosis to cure, these graphic memoirs of sickness by women exhibit a resistive femininity in storytelling [3]. A simple visual presentation of the critical situations in an emergency not only helps the patients cope with their situation and explain it better, but also easily communicates information for patients with language barriers. In mass casualty situations, triage nurses may query patients about their ailments using illustrated symptom checklists, which would enable them to express their feelings clearly and concisely.

Treatment decisions can be made more quickly when patients can point to pictures that depict their level of discomfort, breathing difficulties, or other symptoms. There is evidence detailing that doctors have used medical comic illustrations to explain emergency conditions like stroke due to intracerebral and subarachnoid hemorrhages to the patient's families, when the patient is often unconscious and the need to obtain informed consent and commencement of surgery is of utmost importance. Lengthy explanations by the doctors or nurses often leave the families more perplexed about the prevailing circumstances, and a more lucid explanation in the form of pictures is often easily comprehended by them [4]. Research shows that patients only remember 20% of what doctors say verbally, but that recall can improve up to 50% if there is more written or visual information [5].

In addition to its long-term use in the medical field and emergency departments, dentistry, another field of technical complexity and intimate patient encounters - often performed in high-stress, anxiety-provoking environments - could rely on graphic medicine as well. The integration of graphic medicine in dentistry is not a mere extension but a logical evolution, addressing the discipline's long-standing challenges in patient communication and clinical empathy. In this narrative review, we highlight the increasing popularity of graphic medicine among dental professionals to manage dental fear and anxiety, as well as its role in simplified explanations of dental procedures and postoperative instructions.

Graphic medicine in dentistry

For most patients, dental problems, surgeries, and treatments can be frightening and complicated. Dentists can use graphic medicine, which includes illustrated manuals, info-graphics, and cartoons, to visually explain orthodontic procedures, root canals, fillings, dental implants, and even at-home oral hygiene routines. Visual explanations are frequently easier for patients to understand than text-heavy pamphlets or technical jargon, which lowers anxiety and improves comprehension of dental operations. Dental caries, a prevalent problem among children and adolescents, can be easily controlled by proper oral hygiene maintenance [6]. Parents play an important role in developing awareness about personal hygiene and the care of the child. Thus, in addition to the child, their parents must also be educated and familiarized with dental problems and their management. AI has enhanced this field by easier data visualization and better patient education, using AI tools like Rapid Prototyping, thereby leading to quicker result generation.

## Review

Methods

Review Design and Objective

This narrative review was designed to explore the integration of comics, visual media, and artificial intelligence (AI) in dental and health education. While not a systematic review, the process adhered to structured review methodologies to promote transparency and reproducibility. To improve reporting clarity, the review followed PRISMA (Preferred Reporting Items for Systematic reviews and Meta-Analyses) guidelines for narrative synthesis and methodological transparency. The objective was to identify, analyze, and summarize published literature on the educational, communicative, and clinical applications of graphic medicine and AI in dentistry and healthcare more broadly [7].

Search Strategy

A comprehensive literature search was conducted across four major databases-PubMed, Scopus, Web of Science, and Google Scholar-for studies published between January 1, 2012, and May 31, 2024. The search strategy employed Boolean logic, combining key terms such as “graphic medicine,” “health comics,” “dental comics,” “AI in dentistry,” and “visual health education.” In addition to database searches, the reference lists of included studies were manually screened to identify any additional relevant sources not captured initially.

Eligibility Criteria

Studies were included in the review if they were published in English, peer-reviewed, and discussed the use of comics, illustrations, or AI in health or dental education, communication, or clinical empathy. Exclusion criteria encompassed articles unrelated to health or dental contexts, opinion pieces lacking methodological rigor, and papers that did not provide applied or empirical data to support their claims.

Study Selection and Data Extraction

Titles and abstracts were initially screened by two independent reviewers. Full-text versions were retrieved for articles that met the inclusion criteria or raised uncertainty. Disagreements were resolved through discussion until a consensus was reached. From each included study, data were extracted on author(s), year of publication, study type, population or target audience, nature of the educational or communicative intervention, and major findings or outcomes. The data were synthesized narratively and organized thematically based on patterns emerging from the reviewed literature.

Quality and Risk of Bias Assessment

All included studies underwent critical appraisal using established tools appropriate to their design. The Joanna Briggs Institute (JBI) Critical Appraisal Tools were employed for randomized controlled trials, observational studies, and cross-sectional studies [8]. For narrative reviews, the Scale for the Assessment of Narrative Review Articles (SANRA) was applied [9]. Practitioner-led, art-based, or qualitative studies were assessed for conceptual clarity, methodological rigor, and the coherence between purpose and execution. Two reviewers independently rated each study’s quality and risk of bias as high, moderate, or low.

Reporting of Selection Process

**Figure 1:**
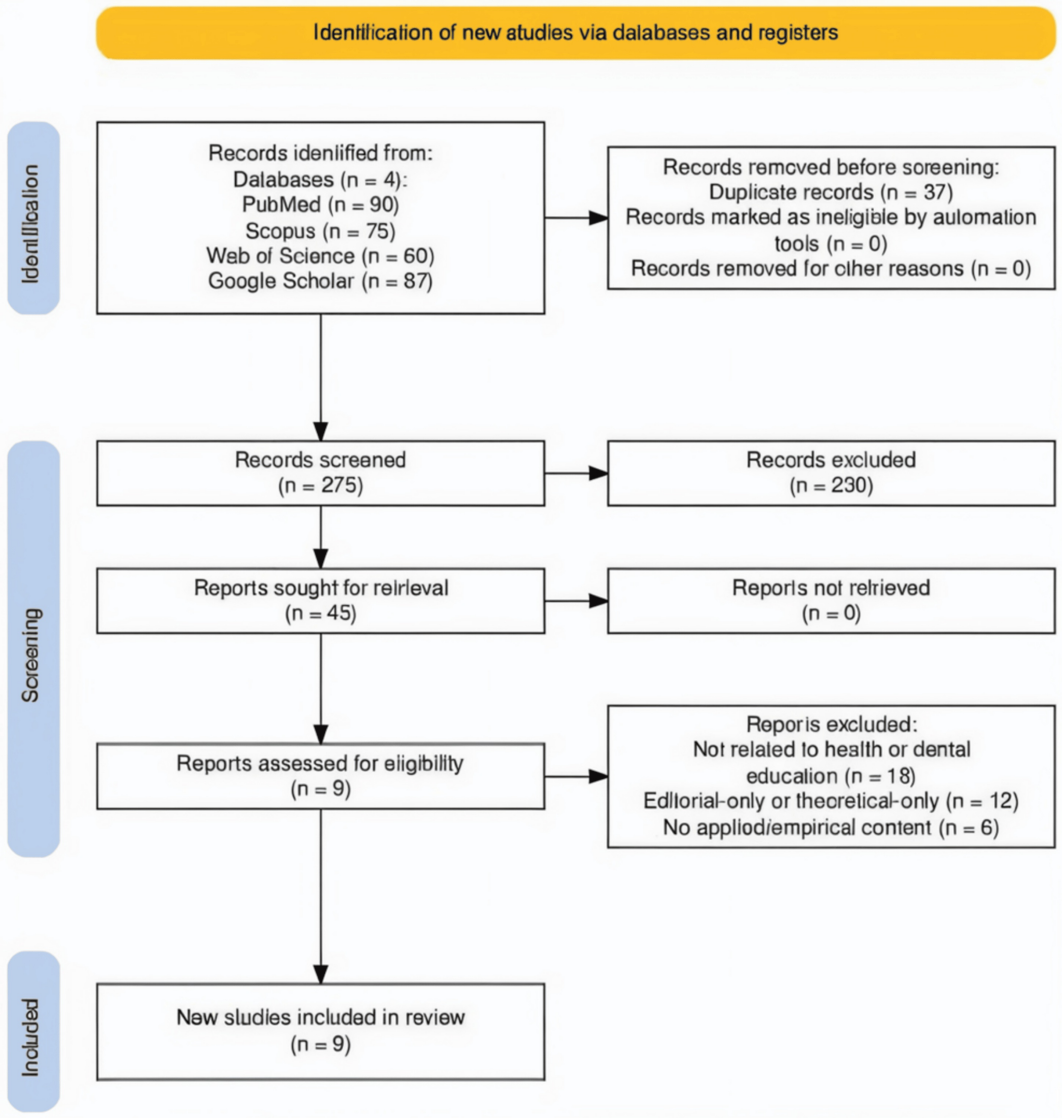
PRISMA 2020 flow diagram depicting study selection This flowchart illustrates the systematic process used to identify, screen, and include relevant studies in the narrative review, based on the PRISMA 2020 guidelines. Records were identified from four electronic databases (PubMed, Scopus, Web of Science, and Google Scholar). After removing duplicates, studies underwent screening and eligibility assessment. A total of nine studies met the inclusion criteria PRISMA: Preferred Reporting Items for Systematic reviews and Meta-Analyses

The process of identifying and selecting studies is illustrated using a PRISMA 2020-compliant flow diagram, generated through the PRISMA 2020 Shiny app and associated R package [7]. This flowchart provides a stepwise account of the number of records identified, screened, excluded (with reasons), and included in the final analysis (Figure 1).

Results

Study Selection

A total of 312 articles were initially identified through searches on PubMed (90), Scopus (75), Web of Science (67), and Google Scholar (80). After removing 37 duplicates, 275 records remained for screening. Title and abstract screening excluded 230 articles that did not meet the inclusion criteria. Full texts of 45 potentially eligible articles were retrieved and assessed. Of these, 36 were excluded for reasons such as lack of relevance to dental or health education (n = 18), editorial or theoretical-only content (n = 12), and the absence of applied findings or empirical data (n = 6). In total, nine articles were included in the final synthesis. The study selection process is detailed in the PRISMA 2020 flow diagram (Figure 1) [7].

Study Characteristics

Among the nine included studies, two were randomized controlled trials [10,11], three were cross-sectional surveys [12-14], two were conceptual or narrative reviews [15,16], one was an observational study [17], and one was a case study describing an arts-based educational intervention [18]. These studies were published between January 2011 and May 2024 and spanned diverse geographic settings, including India, Indonesia, Iran, the United Kingdom, and the United States. Target populations included children, dental patients, parents, dental interns, and practicing clinicians. Educational interventions ranged from patient-facing comics and illustrated consent materials to the use of AI for personalized health education. A summary of the characteristics of each study is presented in Table 1.

**Table 1:** Characteristic of the included studies AI: artificial intelligence: RCT: randomized controlled trial

Study	Year	Country	Study design	Population	Intervention	Key findings
Sosiawan et al. [10]	2020	Indonesia	Cross-sectional study	Elementary school children	Educational comic on dental caries prevention	Comics improved awareness and preventive behavior in children
Elicherla et al. [11]	2024	India	RCT	Pediatric dental patients	Tell-show-do vs. ask-tell-ask	Visual methods reduced dental anxiety more effectively
Oshagh et al. [12]	2011	Iran	Cross-sectional survey	Parents of children	Educational leaflet on orthodontic issues	Increased parental awareness and knowledge
Amanthi and Jeevanandan [13]	2020	India	Cross-sectional study	Parents in urban clinics	Questionnaire on malocclusion awareness	Identified a large awareness gap among parents
Shekhawat et al. [14]	2016	India	Observational	General dental patients	Survey on tooth replacement attitudes	Esthetics is under-prioritized, with low awareness of tooth replacement needs
Al-Jawad [15]	2015	UK	Narrative review	Clinicians and educators	Comics in clinical reflection	Promoted self-reflection and critical engagement in practice
Babaian and Chalian [16]	2014	USA	Case study	Surgical trainees	Comics for teaching thyroidectomy	Improved learner engagement in head and neck surgery education
Waqas et al. [17]	2023	International	Review + case discussion	Pathologists	Generative AI in digital pathology	Demonstrated utility in report generation and workflow improvement
Ahaley et al. [18]	2024	India	Narrative review	Medical writers and educators	Use of ChatGPT in medical writing	Highlighted AI's role in content generation with caveats on accuracy

Thematic Findings

Four major themes emerged from the narrative synthesis. The first was the use of graphic medicine for patient education and informed consent. Several studies described the application of comics and illustrations to enhance patient comprehension and reduce clinical anxiety. For example, Furuno et al. demonstrated how comics were used to explain stroke-related surgeries to families in high-stress emergencies, facilitating informed consent through simplified visual narratives [4]. Babaian and Chalian similarly described the use of comics to teach head and neck surgical procedures [16]. The second theme focused on the application of educational comics in pediatric dentistry. Studies such as those by Sosiawan et al. and Elicherla et al. reported that comics were effective in improving oral hygiene awareness and reducing dental fear among children [10,11]. In both studies, visual tools outperformed traditional written pamphlets in terms of engagement and retention.

The third theme involved parental and community engagement. Oshagh et al. and Amanthi et al. reported that the use of visual educational materials improved parental understanding of orthodontic issues and the importance of early intervention [12,13]. These findings support the utility of comics in extending educational impact beyond the patient to caregivers. The fourth and most emergent theme was the integration of AI in developing and delivering graphic medicine. Waqas et al. highlighted the use of generative models to personalize visual health materials based on patient data [17]. Additionally, Ahaley et al. discussed the role of ChatGPT in automating content generation for educational comics [18].

Quality and Risk of Bias Assessment

Quality appraisal using the JBI tools and the SANRA checklist revealed that four studies were of high quality, three were of moderate quality, and two were of low quality. The overall risk of bias was low to moderate across most included studies. Strengths commonly observed included clearly stated research aims, use of validated tools, and well-documented outcomes. Weaknesses were primarily related to small sample sizes, lack of control groups, or absence of longitudinal data. Full quality ratings and tool-specific scoring are summarized in Table 2 [8,9].

**Table 2:** Risk of bias and quality ratings of included studies JBI: Joanna Briggs Institute; RCT: randomized controlled trial; SANRA: Scale for the Assessment of Narrative Review Articles

Study	Study design	Appraisal tool used	Quality score/max	Risk of bias category
Sosiawan et al. [10]	RCT	JBI RCT checklist	09/10	Low
Elicherla et al. [11]	RCT	JBI RCT checklist	10/8	Low
Oshagh et al. [12]	Cross-sectional survey	JBI checklist	06/8	Moderate
Amanthi and Ganesh [13]	Cross-sectional curvey	JBI checklist	05/8	Moderate
Shekhawat et al. [14]	Cross-sectional survey	JBI checklist	05/8	Moderate
Al-Jawad [15]	Narrative review	SANRA	11/12	Low
Babaian and Chalian [16]	Observational study	JBI checklist	08/9	Low
Ahaley et al. [18]	Narrative review	SANRA	09/12	Moderate
Waqas et al. [17]	Narrative review	SANRA	09/12	Moderate

Discussion

Popularity Among Pediatric Patients

The use of dental comics to explain conditions such as dental caries, their complications, and hygiene management techniques has demonstrated significant value in reinforcing children’s understanding and awareness of oral health [10]. For example, a parent presented with a five-year-old child suffering from extensive caries affecting most of his primary teeth. The child was in considerable pain and discomfort, and the mother reported multiple sleepless nights due to his symptoms. In such cases, dentists typically spend time explaining the disease and treatment options. However, a child experiencing a dental visit for the first time is often overwhelmed by unfamiliar instruments and surroundings, making verbal explanations less effective.

Dental comics can make these situations more manageable for all involved. By presenting information through lucid language and relatable illustrations, comics allow both the child and parent to understand the disease and the procedures involved. This method builds upon the widely used tell-show-do technique in pediatric dentistry, which helps familiarize children with upcoming procedures to reduce anxiety [11]. Comics offer similar benefits, with added visual storytelling that fosters comprehension and cooperation. They effectively familiarize children with what to expect, reducing fear by transforming an intimidating experience into an understandable narrative.

**Figure 2:**
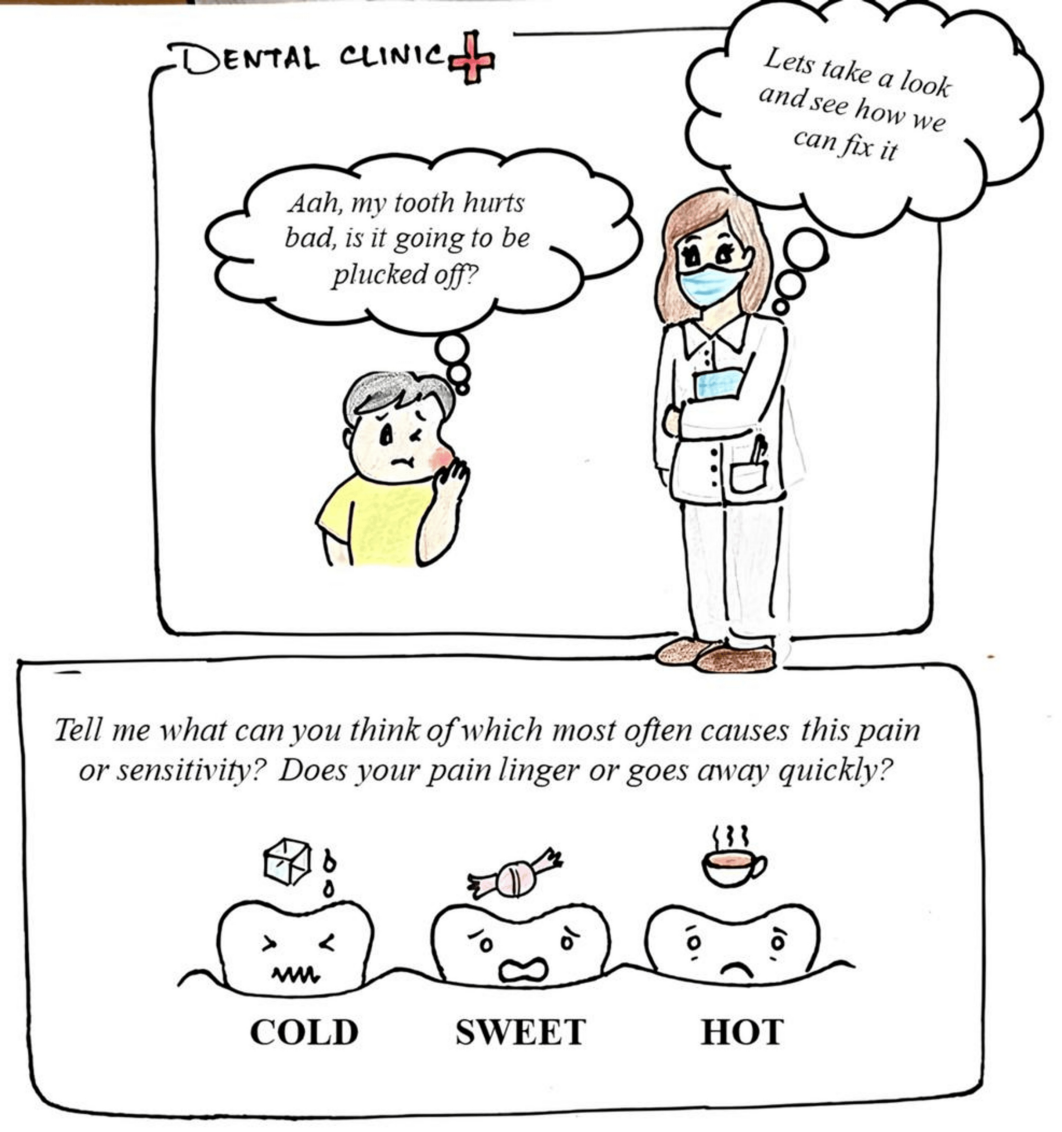
Graphic images of a dental operatory: a young boy walks in anxiously with a toothache and asks the dentist if his tooth would be plucked

**Figure 3:**
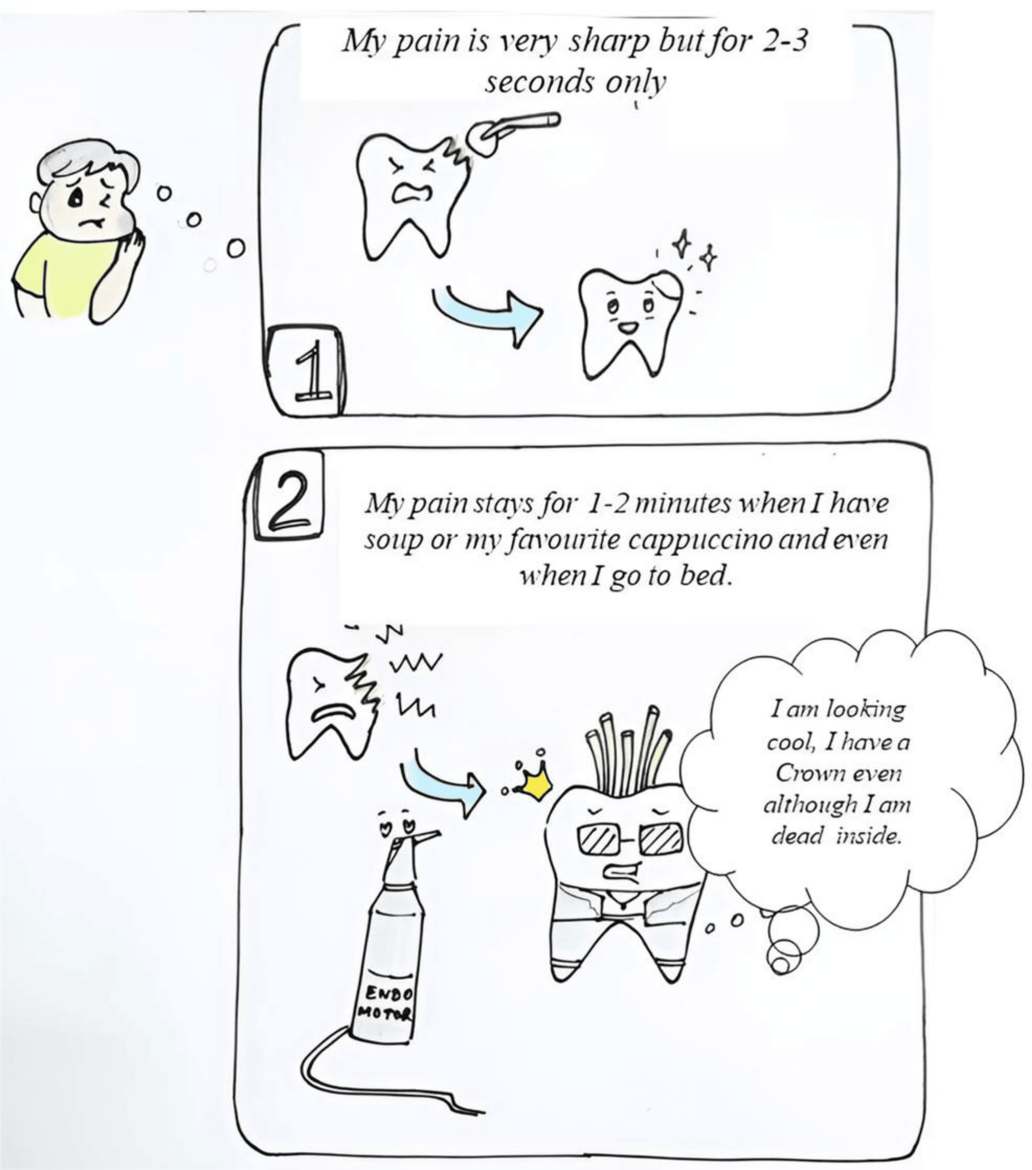
Graphic images that show a dentist explaining whether the tooth will be restored with a filling or a root canal treatment in the child's language

Figures 2-3 illustrate such a scenario: a child suffering from dental pain navigating a dental visit with the help of visual tools and clear storytelling. Figure 2 portrays the child’s initial anxious encounter, while Figure 3 shows the dentist calmly explaining whether the tooth will need a filling or a root canal treatment in simple, child-friendly terms.

Awareness About Teething and the Required Intervention

The period of primary tooth exfoliation is a critical phase in a child’s oral development. It is during this time that parents should be informed about the potential consequences of neglecting primary dental care, including the development of dentofacial malocclusions and harmful oral habits. Topics such as the correct timing for orthodontic assessments, the risks of untreated malocclusion, and the importance of early intervention must be clearly communicated by family dentists.

Children undergoing orthodontic treatments, such as braces or retainers, are more likely to comply when they understand the rationale behind the procedures. Comics tailored for this purpose can simplify complex orthodontic concepts and make them more relatable. By embedding these ideas into familiar, narrative-driven visual content, orthodontic practices can create educational materials that resonate with both children and their parents. This not only enhances treatment adherence but also nurtures a culture of early diagnosis and preventive dental care.

As seen in Figures 2-3, where the young patient interacts with the dentist about his toothache and learns about possible treatment options like fillings or root canals, comics help both children and adults visualize clinical procedures and participate more confidently in treatment planning.

Management of Malaligned or Missing Teeth

During the mixed dentition years, parental awareness of their child’s dental alignment plays a key role in early intervention. Yet, in many cases, parents remain either uninformed or indifferent to issues such as malocclusion. A study from India found that approximately 83% of parents were unaware of their child’s malalignment or any contributing oral habits [13]. Even when dentists provide detailed explanations, many families struggle to grasp the clinical implications or visualize corrective procedures.

In clinical practice, patients with multiple missing teeth often present without major complaints. Upon further questioning, it becomes evident that many prioritize adapted functional efficiency over aesthetics, a trend particularly noticeable in lower-income or rural populations. This highlights the need for effective visual communication to raise awareness about the importance of tooth replacement and the consequences of neglecting missing teeth.

Illustrative aids, such as schematic diagrams or comic-based visuals, can be highly effective in such scenarios. A long verbal explanation of prosthetic options is unlikely to influence behavior unless patients can clearly visualize the relevance of a single missing tooth to overall dental function. When treatment options are explained using illustrations or comics, patients and their families tend to engage more positively with the treatment plan. These visual tools can greatly enhance rapport and support informed decision-making, reinforcing the patient’s role in maintaining oral health.

Popularity Among Medical Students

Graphic medicine is also gaining traction in medical and dental education, where it serves as a valuable tool for simplifying complex academic and clinical scenarios. Comics present multi-layered clinical experiences in an accessible format, helping students correlate theoretical knowledge with real-life patient care. This narrative-based visual learning enhances not only retention but also the emotional and ethical awareness necessary for empathetic practice.

The concept of “haptic overlap”-the cognitive link between drawing and manual skill-underscores the role of visual learning in clinical training. For centuries, anatomical illustrations have supported surgical education, with drawing used not only to learn but also to internalize spatial relationships and procedural steps. This kinesthetic element of learning may activate similar neural pathways involved in clinical manipulation and procedural execution [14,15].

Michael J. Green’s graphic narratives, for example, depict the journey of medical residents navigating uncertainty, emotional fatigue, and critical thinking during their formative years. These narratives emphasize that medical education is not a linear accumulation of facts, but a complex, reflective process requiring empathy, intuition, and judgment [19]. As a medium, comics support this by allowing students to encounter the realities of patient care-including ambiguity and emotional nuance-in a controlled, digestible, and impactful format.

Graphic Medicine Over Conventional Leaflets

Traditional patient information leaflets often present factual content in a neutral, text-heavy format. While they deliver accurate information, their impact is frequently limited by low engagement, especially among patients with lower health literacy or emotional resistance to clinical topics. In contrast, comics present health information through relatable narratives and visual storytelling, which can evoke empathy and foster emotional understanding.

This narrative immersion allows patients to identify with characters who mirror their own experiences or concerns, thereby making health information more approachable. Rather than abstract instruction, comics convey the lived experience of illness, capturing emotional nuances such as fear, hope, and confusion in a way that fosters self-awareness and understanding. The storytelling format also invites family members to share in the learning experience, promoting shared decision-making and reducing anxiety.

Research supports the effectiveness of comics as an educational medium. Studies show that visual narratives can increase knowledge retention, stimulate discussion, and improve emotional processing of difficult health topics [20]. For example, comics have been successfully used in public health campaigns addressing smoking among adolescents, where their visual format and character-driven messaging proved more persuasive than conventional methods [21].

Through these mechanisms, graphic medicine serves as more than a communication aid-it becomes a tool for empathy-building and patient empowerment.

Future Scope

As graphic medicine continues to evolve, its integration with emerging technologies is opening new frontiers in healthcare communication. AI and machine learning are now being used to augment medical education, patient interaction, and research visualization. Traditionally, convolutional neural networks (CNNs) have been effective in image processing tasks such as pathology slide analysis, yet their limitations include dependency on large, annotated datasets and reduced generalizability across new samples or techniques. The introduction of foundation models and generative AI tools in the 2020s addresses many of these challenges by offering in-context learning, adaptability, and self-correction [17].

In pathology, for instance, generative AI models trained on large datasets can support image interpretation, diagnosis, and report generation-improving both accuracy and efficiency. Similarly, AI-driven platforms like ChatGPT can assist in drafting medical narratives from patient data or clinician inputs, which illustrators can transform into comics tailored for specific educational or counseling needs. While these tools can streamline content development, human oversight remains essential to ensure medical accuracy, ethical standards, and contextual appropriateness [18].

Further, applications such as DALL·E offer healthcare professionals a means to generate custom illustrations from text descriptions, allowing for the visualization of abstract or complex medical concepts. These tools enhance accessibility and engagement, particularly in communities with language barriers or limited health literacy. By combining narrative storytelling with personalized visual content, graphic medicine-augmented by AI-has the potential to improve patient understanding and inclusivity in care delivery [18].

The global market for comic books and visual storytelling media is expanding rapidly, bolstered by the widespread adoption of digital platforms. This trend suggests a rising opportunity for healthcare providers to leverage graphic content in patient education, public health campaigns, and digital health interventions. The growing accessibility and appeal of comic-based media present a promising avenue for creating impactful, inclusive, and emotionally resonant health communication strategies [22].

## Conclusions

Graphic medicine has emerged as a versatile and impactful tool in dental and medical communication, offering innovative ways to simplify complex information and foster empathy across diverse patient populations. Its effectiveness is evident in pediatric dentistry, orthodontic counseling, prosthetic awareness, and professional training, where visual narratives enhance comprehension, reduce anxiety, and support behavioral change. The integration of comics into health education strengthens patient-clinician rapport and promotes participatory care. As emerging technologies like AI and generative design tools are introduced to the field, the scope of graphic medicine expands further-enabling personalized, accessible, and engaging health content. When applied with clinical oversight, this medium holds significant potential to bridge gaps in communication, improve treatment outcomes, and create a more empathetic and inclusive healthcare experience.
